# Platelet counts and outcome in the pediatric intensive care unit

**DOI:** 10.4103/0972-5229.43678

**Published:** 2008

**Authors:** Shruti Agrawal, Anil Sachdev, Dhiren Gupta, Krishan Chugh

**Affiliations:** **From:** Pediatric Intensive Care, Sir Ganga Ram Hospital, New Delhi, India

**Keywords:** Coagulopathy, mortality, pediatric intensive care, platelets, prognosis, thrombocytopenia

## Abstract

**Objectives:**

Thrombocytopenia is commonly observed in critically ill patients. This study was undertaken to evaluate the variation in platelet counts and the risk factors associated with thrombocytopenia and mortality in pediatric intensive care patients. In addition, prognostic value of platelet counts for outcome in pediatric intensive care unit was studied.

**Study Design:**

Prospective, observational cohort analysis.

**Setting:**

8- bedded pediatric intensive care unit of a tertiary care teaching hospital.

**Patients:**

All consecutively admitted patients (n=138) staying in the pediatric intensive care unit (PICU) for at least 48h over a 7 months period were studied.

**Measurements and Main Results:**

Thrombocytopenia was defined as platelet counts <150.0/nL. Median 1^st^ day Pediatric Risk of Mortality Score (PRISM) was 5 (range 0-30) and median ICU stay was 4 days (range 2-98 days). Twenty five percent patients had at least one episode of thrombocytopenia during the stay. Twenty percent of these patients had thrombocytopenia on admission and rest (80%) developed it during the PICU stay. Seventy one percent (19) of the patients developed thrombocytopenia by fourth day of admission. Patients with PICU acquired thrombocytopenia had statistically significant lower baseline, nadir and 4th day platelet counts and a significantly higher drop in platelet counts (56% vs. 6% *P*<0.001) as compared to non thrombocytopenic patients. PRISM score, long PICU stay, sepsis, coagulopathy, and creatinine levels were significantly associated with occurrence of thrombocytopenia. Patients with thrombocytopenia had higher probability of bleeding (34% vs. 15%, *P*=0.01). Higher platelet counts on admission were associated with significantly reduced risk of thrombocytopenia (*P*=0.00) Baseline, nadir and day-4 platelet counts, presence of thrombocytopenia on admission, sepsis, coagulopathy and a higher mean PRISM score on univariate analysis were significantly associated with mortality. Leucopenia or leucocytosis, thrombocytopenia and coagulopathy were found to significantly affect outcome. Drop in platelet counts was found to have slightly higher discriminative value for mortality prediction than PRISM on the ROC curve. The survivors had higher platelet counts throughout the PICU stay and after an initial fall in platelet counts in the PICU showed a significantly higher rise in the platelet counts in the following days than the non-survivors.

**Conclusions:**

Thrombocytopenia is common in PICU. Patients requiring cardiopulmonary resuscitation or with circulatory shock, coagulopathy, sepsis and with more severe disease have higher risk of developing thrombocytopenia. Thrombocytopenic patients have a higher risk of bleeding. Drop in platelet counts >27% and thrombocytopenia were independently related to mortality. Serial measurements of platelet counts are better predictors of pediatric intensive care outcome than one-time values. Any drop in platelet counts even without thrombocytopenia needs an urgent and extensive evaluation.

## Introduction

The platelet count, which was related only with bleeding and hemostasis, is now considered to be a predictor of outcome in the ICU setting as an independent parameter.[1] It is found to be as good a predictor as the various mortality scores used in the ICU.[2] This is attributed to the important role played by platelets in the inflammatory process apart from their role in thrombus formation.[3]

Thrombocytopenia is one of the commonest laboratory abnormalities encountered in ICU and the reported incidence varies from 13 to 58% in various studies.[4–7] Various causes have been identified for the occurrence of thrombocytopenia, like presence of disseminated intravascular coagulation, immune mechanisms, reduced production, increased consumption or abnormal sequestration of platelets or a combination of these.[8,9]

The advantage of using platelets as a predictor of ICU outcome, is the dynamic nature of daily platelet counts which takes the disease progression into account in contrast to various mortality scores which use only the worst parameters within first 24h after admission or at admission.[1] Various adult studies have shown an initial decrease in platelet count followed by increase, but no such study has been carried out in critically ill children.[10–12] This reflects the initial inflammatory phase when the patient is admitted to the ICU with the increase in platelet counts predicting onset of anti-inflammatory action. Hence, we undertook this study to establish the variations in platelet counts in the critically ill pediatric patients, the factors associated with thrombocytopenia and outcome in the Pediatric Intensive Care Unit (PICU), the factors associated with outcome in thrombocytopenic patients and the prognostic value of serial platelet counts in the PICU.

### Design

This study was a prospective, observational cohort analysis. It was undertaken in the Pediatric Intensive Care Unit of a tertiary care, teaching hospital in New Delhi, India over a period of 7 months from 1^st^ January 2003 to 31^st^ July 2003.

## Materials and Methods

All consecutively admitted patients staying for 48h or more in the PICU over a period of 7 months were included in the study. The patients were followed-up prospectively until they left the PICU or died. Besides patients' demography, source of admission, primary diagnosis, pediatric risk of mortality score (PRISM), presence or absence of sepsis, bleeding, use of central venous or arterial lines and mechanical ventilation were recorded. Laboratory data collected at admission included complete blood counts (CBC), C- reactive protein (CRP), blood urea nitrogen (BUN), serum creatinine, serum bilirubin, serum lactate and coagulation profile. These were also repeated with the occurrence of thrombocytopenia. Daily platelet counts were recorded in each patient. If any patient had platelet counts done more than once in 24h, the lowest value was recorded for analysis. No informed consent was taken, as the study was purely observational. The hospital ethics and review board's approval was taken before undertaking the study.

### Definitions

Thrombocytopenia was defined as a platelet count of <150.0/nL. It was categorized depending on the severity as mild, moderate, severe or very severe on the basis of platelet counts below 150.0/nL, 100.0/nL, 50.0/nL or 20.0/nL, respectively. Correction of thrombocytopenia was defined when the platelet counts normalized after falling below 150.0/nL. The lowest platelet count for any patient was considered as nadir platelet count. A drop in platelet count was taken as a difference between the admission platelet counts and the nadir platelet counts for each patient. Intensive care unit acquired thrombocytopenia was considered when the patient was admitted with normal platelet count, which dropped below 150.0/nL subsequently during the PICU stay.

Sepsis was defined in patients with documented or assumed infection in presence of positive acute phase reactants and total leukocyte counts (TLC); (as defined by the American College of Chest Physicians and Society of Critical Care Medicine).[13] Coagulopathy was defined when the APTT was 1.5 times the normal reference range for the laboratory with an associated increase in INR more than 1.5. Bleeding was defined as an episode resulting in a drop in hemoglobin of >2 g/dL within 24h, episodes requiring local tamponade or transfusions within 24h, and any intracranial hemorrhage. Multiple bleeding events at the same site were counted only once for each patient. Circulatory failure was defined as the need for vasoactive drugs for at least one hour (>5μg/kg/mt dopamine/dobutamine or any dose of epinephrine/nor-epinephrine).

### Statistical analysis

Statistical Analysis was done using STATA 9.1 (College Station, Texas, USA). Data were presented as number (%) or median (range) as appropriate. Categorical variables were compared with the outcome variable using Chi-square or Fisher Exact test. Receiver Operating Characteristic Curve analysis was used to find the cut-off value for PRISM score in relation to the outcome of thrombocytopenia. Simple Logistic regression followed by forward stepwise multiple logistic regression with the significance level 0.10 for removal and of 0.05 for addition to the model to identify the factors associated with thrombocytopenia. The p value less than 0.05 was considered statistically significant.

## Results

Out of the 268 total admissions in PICU during the study period, 138 patients who remained in the unit for at least 48h were included in the study [Table 1].

**Table 1:** Demographic Data of the patients admitted to the PICU (n= 138)

Parameter	Total
Age, median months (range)	32 (1–192)
Gender ratio (M: F)	1.76: 1
PRISM score (median (range)	5 (0–30)
Source of admission	
Emergency room	110
Hospital ward	18
Operation theatre	10
Admission category	
Neurological	33 (23.9%)
Monitoring	25 (18%)
Circulatory failure	24 (17.3%)
Respiratory failure	23 (16.6%)
Cardiopulmonary resuscitation	16 (11.5%)
Hepatic failure	12 (8.6%)
Renal failure	6 (4.3%)
ICU stay median (range) days	4 (2 – 98)
< 7 d	99 (71%)
7–14 d	19 (13.7%)
> 14 d	20 (14.5%)
Mechanical ventilation	33 (23.9%)
Central venous catheter	76 (55%)
Arterial catheter	63 (45.6%)
Sepsis	49 (35.5%)
Coagulopathy	21 (15.2%)
Bleeding	27 (19.5%)
Transfusion	30 (21.7%)
ICU mortality	10.9%

### Platelet counts

At least one episode of thrombocytopenia was seen in 35 patients (25%), of these 8 patients had thrombocytopenia on admission; the rest developed it during the course of PICU stay. Mild, moderate, severe, and very severe thrombocytopenia was present in 8.6%, 42.5%, 28%, and 20% of patients respectively. Seventy one percent of patients had PICU acquired thrombocytopenia by fourth day of admission (range: 2-5 d). About 59% of the patients staying in PICU for more than 7 days had at least one episode of thrombocytopenia. Patients with PICU acquired thrombocytopenia had statistically significant lower baseline, nadir and day- 4 platelet counts corresponding to a 56% drop from median baseline values vs. 6% in non-thrombocytopenic patients (*p* value <0.001). Platelet counts dropped more than 10% in 66 patients (48%). Platelet counts had normalized by the time of discharge or death in 65% of thrombocytopenic patients.

### Factors associated with thrombocytopenia

Age, gender, source of admission, CRP and total leukocyte counts (TLC), and mechanical ventilation had no significant correlation with the development of thrombocytopenia.

Cardiopulmonary resuscitation (CPR) and circulatory failure as admission category were found to be significant factors for the development of thrombocytopenia (5/103 vs. 11/35 patients, *P* < 0.00 and 14 of 103 vs. 10 of 35, *P* = 0.002, respectively). The other factors found associated with development of thrombocytopenia are shown in Table 2. A higher platelet count on admission was associated with reduced risk of thrombocytopenia (*P*-0.00, OR- 1.00 for every 100.0/nL, 95% CI: 1.00007 – 1.00016).

**Table 2:** Clinical parameters associated with thrombocytopenia

Variable	Thrombocytopenia		*P* value
		
	Yes (n= 35)	No (n= 103)	
PRISM score^a^			
< 8	6 (17)	74 (71.8)	0.00
> 8	29 (82. 8)	29 (28)	0.00
Admission category^b^			0.00
CPR	11 (31.4)	5 (4.8)	
Circulatory Failure	10 (28.6)	14 (13.6)	
Admission Source^b^			0.23
Emergency Room	24 (88.5)	86 (83.4)	
Operation Theatre	3 (8.5)	7 (6.8)	
Hospital Ward	8 (22.8)	10 (9.7)	
Surgical procedure^b^	12 (34)	21 (20.3)	0.09
Coagulopathy^b^	15 (42.8)	6 (5.8)	0.00
Central venous catheter^b^	35 (100)	41 (39.8)	0.00
Arterial catheter^b^	29 (82.8)	34 (33)	0.00
Sepsis^b^	23 (65.7)	26 (25)	0.00
Bleeding^b^	12 (34)	15 (14.5)	0.01
Transfusion^b^	19 (54)	11 (10.6)	0.00
TLC (/mm3)^b^	0.098		
> 15000	9 (25.7)	12 (11.6)	
< 4000	1 (2.8)	8 (7.7)	
BUN (> 20 mg/dl)^b^	6 (17)	6 (5.8)	0.04
S. Creatinine (> 1.2 mg/dl)^b^	5 (14)	4 (3.8)	0.031
Bilirubin (> 1 mg/dl)^b^	19 (54.2)	22 (21)	0.00
CRP (> 6 mg/dl)^b^	22 (62.8)	46 (44.6)	0.063
ICU stay (days)^b^			0.00
< 7 days	8 (22.9)	91 (88.3)	
7-14 days	9 (25.7)	10 (9.7)	
> 14 days	18 (51.4)	2 (1.9)	
Lactate (>2 mg/dl)	31 (96.8)	87 (98.8)	0.45

a Data represented as median with 25th and 75th quartiles in the parentheses

b Data presented as no. of patients with percentage in parentheses

On forward stepwise multiple regression analysis, length of PICU stay, PRISM, Sepsis, coagulopathy and creatinine were the only factors found significantly associated with thrombocytopenia [Table 3].

**Table 3:** Significant Risk Factors associated with thrombocytopenia (regression analysis)

Variables	OR (95% Confidence Interval)	
	
	Unadjusted	Adjusted
Sepsis	5.7 (2.5- 12.9)	6.22 (1.4- 27.9)
Coagulopathy	12 (4.2- 35.1)	14.8 (2.4- 93.2)
Arterial Catheter	8.6 (3.3- 22.7)	
Bleeding	3.1 (1.3- 7.4)	
Transfusion	9.9 (3.9- 24.7)	
BUN	3.3 (1- 11)	
Creatinine	4 (1.04- 16.3)	17.4 (1.3- 233.6)
CRP	2.1 (0.9- 4.6)	
Bilirubin	4.4 (1.9- 9.9)	
PRISM	12.3 (4.6- 32.8)	9.2 (2.02- 42)
ICU stay		12.5 (4.2- 37.4)

### Outcome and factors associated with mortality

There was a significant association between mortality and the presence of sepsis, coagulopathy, and higher mean PRISM score, leucocytosis, leucopenia, hyperbilirubinemia, and CRP levels. There was no difference in mortality with age, gender, source of admission and diagnosis, and the use of mechanical ventilation, arterial or venous catheters or bleeding [Table 4].

**Table 4:** Clinical parameters associated with outcome

Variable	Survivors	Non survivors	*P* value
PRISM score^a^			0.009
< 8	76 (61.8)	4 (26.7)	
> 8	47 (38.2)	11 (73.3)	
Admission category^b^			
CPR	13 (10.6)	3 (20)	0.28
Admission Source^b^			0.13
Emergency Room	99 (80.4)	11 (73.3)	
Operation Theatre	9 (7.3)	1 (6.7)	
Hospital Ward	15 (12.2)	3 (20)	
Surgical procedure^b^	29 (23.6)	4 (26.7)	0.79
Coagulopathy^b^	14 (11.3)	7 (46.7)	0.00
Central venous catheter^b^	57 (46.3)	9 (60)	0.31
Sepsis^b^	40 (32.5)	9 (60)	0.03
Bleeding	23 (18.7)	4 (26.7)	0.46
Transfusion^b^	23 (18.7)	7 (46.7)	0.01
TLC (/mm3)^b^			0.017
> 15000	15 (12)	6 (40)	
< 4000	8 (6.5)	1 (6.7)	
BUN (> 20 mg/dl)^b^	10 8	2 (13.3)	0.5
S. Creatinine (>1.2 mg/dl)^b^	8 (6.5)	1 (6.7)	0.98
Bilirubin (>1 mg/dl)^b^	33 (26.8)	8 (53.3)	0.034
CRP (> 6 mg/dl)^b^	57 (46.3)	11 (73.3)	0.048
ICU stay^b^			0.30
< 7 days	90 (73)	9 (60)	
7- 14 days	15 (12)	4 (26.7)	
> 14 days	18 (14.6)	2 (13.3)	
Lactate (> 2 mg/dl)^b^	103 (98)	15 (100)	0.59

a Data represented as median with 25th and 75th quartiles in the parentheses

b Data presented as no. of patients with percentage in parentheses

**Figure 1:**
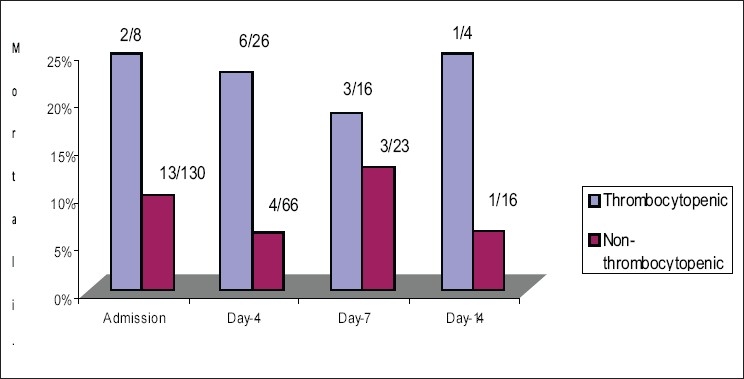
Mortality Rate in thrombocytopenic vs. non-thrombocytopenic patients on admission, day-4, day-7 and day-14

**Figure 2:**
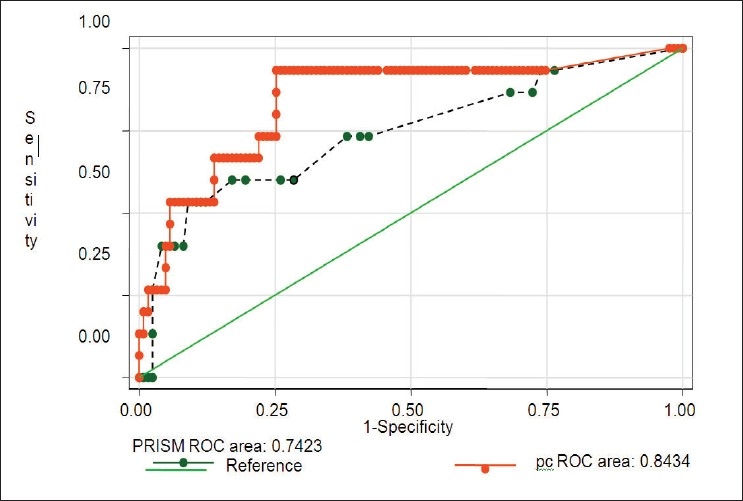
Receiver operating characteristic curve for drop in platelet counts (pc- solid line), PRISM (interrupted line)

The mortality showed a significant association with platelet counts. The presence of thrombocytopenia was associated with increased mortality (33% *vs* 66.6%, *P* - 0.00). The mortality was significantly higher with lower platelet counts on day-1, day-4 and lower nadir platelet counts irrespective of the presence of thrombocytopenia. For the study population, platelet counts were lower in the non-survivors than survivors throughout the ICU stay. The admission platelet counts were lower in non-survivors than in survivors (*P* - 0.006) [Figure 1]. Thirty-nine (28.3%) patients stayed in the PICU for > 7 days (median stay 15 days, range 7-98 days) and in this group the mortality was 15.4%. Platelet counts decreased significantly in the initial 4-5 days of PICU stay in both survivors and non-survivors and gradually increased to near admission value by the end of first week [Figure 1]. In non-survivors the platelets counts did not rise significantly after the first week as compared to the survivors. A drop of > 27% from the baseline platelet counts increased the mortality (Receiver operator curve); survivors had a mean drop of 8% (range 0-29.6%) compared to 83% (range: 27-85%) in non-survivors from the baseline (*P*<0.00). Drop in platelet counts was found to have slightly higher discriminative value for mortality prediction than PRISM score on the receiver operating characteristic curve (ROC) curve. The area under the curve of PRISM ROC was 0.73 and that of the drop in platelet count was 0.84. This difference was not statistically significant [Figure 2]. On day 4, 26 (28.3%) patients had thrombocytopenia and the mortality in this group was 23.1% as compared to 6.1% in nonthrombocytopenic patients (*P* = 0.02). Normal platelet counts on admission were associated with better survival than normal platelet counts on day 4 and day 7 (*P*<0.05).

On forward stepwise multi-regression analysis, leucopenia, leucocytosis, thrombocytopenia and coagulopathy were found to be the risk factors independently associated with mortality [Table 5].

**Table 5:** Significant Risk Factors for Outcome (Regression Analysis)

Variables	OR (95% Confidence Interval	
	
	Unadjusted	Adjusted
Sepsis	5.7 (2.5- 12.9)	
TLC (< 4000/mm3)	1.56 (0.17- 14.1)	2.08 (0.18- 23.9)
TLC (> 15000/mm3)	5 (1.5- 16.4)	4.3 (1.14- 16.5)
Coagulopathy	6.8 (2.14- 21.67)	3.5 (0.8- 14.5)
Transfusion	3.8 (1.25- 11.5)	
BUN	3.3 (1- 11)	
CRP	3.18 (0.96- 10.5)	
Bilirubin	3.11 (1.1- 9.27)	
PRISM	4.4 (1.34- 14.7)	
Thrombocytopenia	7.84 (2.45- 25)	4.15 (1.05- 16.3)

## Discussion

The present study has demonstrated an association of a drop in platelet counts during PICU stay with the outcome. At least one episode of thrombocytopenia occurred in 25% of patients. The incidence of thrombocytopenia and PICU acquired thrombocytopenia is comparable to one adult study,[1] but less in our study as compared to similar recent study by Strauss *et al*,[4] where 44% of the patients acquired thrombocytopenia while in the ICU. The incidence of thrombocytopenia has ranged from 13-58% in various studies.[4–7] The difference in the ranges can be explained by the differences in study population and different inclusion criteria and definitions used in various studies. The incidence of platelet counts <100.0/nL was 23.2%, which is comparable to other studies; 22% in a neonatal ICU,[15,16] 23% in a medical ICU,[14] 22% in medical-surgical ICU[1] and 21% in a non-coronary medical ICU.[4] The incidence of thrombocytopenia was significantly correlated with the baseline platelet counts, nadir platelet counts and a drop in platelet counts as has been evident in other similar studies.[1,4]

Sepsis was also found to have an association with thrombocytopenia, 65 % of the patients with sepsis had thrombocytopenia (23 of 49), which is comparable to other adult studies.[1,5,14] Coagulopathy was another factor associated with thrombocytopenia, which is comparable to the significant association of DIC in the evolution of thrombocytopenia found by Strauss *et al.*[4] This has been explained by the intravascular destruction of platelets, which accompanies the activation and consumption of coagulation factors and is described as the leading cause of thrombocytopenia in critically ill patients.[14]

Cardio-pulmonary resuscitation has also been quoted as a risk factor for development of thrombocytopenia[4] and a similar association was found in the present study (11 of 16, 31.4%). The other admission category, which was found, significantly associated with thrombocytopenia was circulatory failure (peripheral or cardiovascular) (14 of 103 vs. 10 of 35, *P* = 0.002). The association of shock with thrombocytopenia is well established.[1] A higher initial PRISM score and disturbed biochemical markers in the form of elevated blood urea nitrogen, serum creatinine, bilirubin, lactate, which identify sicker patients, were also predictive of thrombocytopenia. Invasive intravascular catheters (arterial or venous) and mechanical ventilation have been described in the literature as an independent risk factor for development of thrombocytopenia, though this may only reflect the disease severity and local ICU preferences.[4,6] In our study, there was no such association observed. This may be due to small patient number.

Patients who stayed in the PICU for a longer period had a higher incidence of thrombocytopenia reflecting the disease severity necessitating prolonged stay and increased risk of intensive care acquired sepsis. The increased risk of thrombocytopenia with prolonged ICU stay is also seen in adult studies.[1,4,11,14]

The present study clearly shows significantly higher mortality in thrombocytopenic patients especially low platelet counts on admission, in first week of PICU stay and failure of platelet counts to return to normal. The study further shows a bimodal pattern of platelet counts with the counts falling to a nadir in the initial 3-4 days of PICU admission followed by increase to near the admission value by the end of first week. This rise in platelet count after an acute decrease occurs because of the bone marrow response and is a common observation in other studies.[5,6,11,12] A drop in platelet counts of > 27% was associated with significantly higher mortality irrespective of the actual counts. This finding is comparable with a recent adult study with > 30% drop in platelet counts.[4]

Cardio-pulmonary resuscitation was not found to be significantly associated with mortality as has been found in other studies.[4] This can probably be due to the fact that very few patients needed cardio-pulmonary resuscitation and a substantial number of these were performed within the hospital. Furthermore, the ROC curve for PRISM and drop in platelet counts showed a slightly higher discriminative value of drop in platelet counts for mortality, which is similar to what was found in certain adult studies.

The mortality in thrombocytopenic patients was significantly higher in patients with prolonged PICU stay, lower nadir and D-4 platelet counts and with disease severity markers. The mortality in this group was not found associated to the admission platelet counts signifying that the predictive power of low platelet counts does take the disease progression in account.

There are no similar pediatric studies to compare the data with, though there are a few neonatal studies done on the incidence and prognostic value of low platelet counts on admission.[15,16] The studies on thrombocytopenia in pediatric ICU are few and the ones which have been done have very different objectives than our study rendering it difficult to compare the present work with any pediatric study.[10]

The present study had its own limitations. Besides small sample size, various confounding factors are present at any given point of time in critically ill children, which cannot be controlled. Many pre-existing conditions and drugs in use may influence the platelet counts, which were not studied in this cohort. The limited number of patients in certain groups does not allow great precision in the estimation of odds ratio and this may have missed some important risk factors. So the results need to be validated in a larger cohort. Bleeding can be both a risk factor and cause for thrombocytopenia and this was not elaborated sufficiently in the present study, though most of the patients had bleeding secondary to thrombocytopenia rather than vice-versa. The study is not powered to actually work out the cause and effect and can only suggest an association between various factors.

## Conclusions

Platelet counts < 150.0/nL is at least as common in PICU as in adult intensive care. Thrombocytopenic children have higher incidence of bleeding, longer ICU stay and a higher mortality. Probability of PICU acquired thrombocytopenia is higher in patients requiring cardiopulmonary resuscitation, having circulatory failure, sepsis, coagulopathy and higher admission PRISM score and patients having other parameters defining increased disease severity.

Serial platelet counts are easily available markers of disease progression. A drop in platelet counts irrespective of thrombocytopenia is an unfavorable prognostic marker and is associated independently with the mortality.

Similar studies are required with larger number of patients in the pediatric age group to further consolidate the present study's findings.
